# Measurement of Circulating 1,25-Dihydroxyvitamin D: Comparison of an Automated Method with a Liquid Chromatography Tandem Mass Spectrometry Method

**DOI:** 10.1155/2016/8501435

**Published:** 2016-04-05

**Authors:** Armin Zittermann, Jana B. Ernst, Tobias Becker, Jens Dreier, Cornelius Knabbe, Jan F. Gummert, Joachim Kuhn

**Affiliations:** ^1^Clinic for Thoracic and Cardiovascular Surgery, Heart and Diabetes Center North Rhine-Westphalia, Ruhr University Bochum, 32545 Bad Oeynhausen, Germany; ^2^Institute for Laboratory and Transfusion Medicine, Heart and Diabetes Center North Rhine-Westphalia, Ruhr University Bochum, 32545 Bad Oeynhausen, Germany

## Abstract

*Background*. The clinical relevance of circulating 1,25-dihydroxyvitamin D (1,25(OH)_2_D) is probably underappreciated, but variations in the measurement of this difficult analyte between different methods limit comparison of results.* Methods*. In 129 clinical samples, we compared a new automated assay with a commercially available liquid chromatography tandem mass spectrometry (LC-MS/MS) kit.* Results*. Median (interquartile range) 1,25(OH)_2_D concentrations with the automated assay and the LC-MS/MS method were 26.6 pg/mL (18.5–39.0 pg/mL) and 23.6 pg/mL (16.1–31.3 pg/mL), respectively (*P* = 0.001). Using the method-specific cut-offs for deficient 1,25(OH)_2_D levels (<20 pg/mL for the automated assay and <17 pg/mL for the LC-MS/MS method), the percentage of patients classified as 1,25(OH)_2_D deficient was 28.7% and 27.1%, respectively. However, concordance between the two methods for deficient levels was only 62% and the concordance correlation coefficient was poor (0.534). The regression equation resulted in an intercept of −1.99 (95% CI: −7.33–1.31) and a slope of 1.27 (95% CI: 1.04–1.52) for the automated assay. The mean bias with respect to the mean of the two methods was −3.8 (1.96 SD: −28.3–20.8) pg/mL for the LC-MS/MS method minus the automated assay.* Conclusions*. The two methods show only modest correlation and further standardization is required to improve reliability and comparability of 1,25(OH)_2_D test procedures.

## 1. Introduction

Vitamin D deficiency is regarded as a worldwide health problem that probably affects not only musculoskeletal health but also a wide range of acute and chronic diseases [1]. Therefore, the assessment of vitamin D status and the treatment of vitamin D deficiency are considered to be important issues of interest to public health [2–4].

Although 1,25-dihydroxyvitamin D (1,25(OH)_2_D) is the active, hormonal form of vitamin D, its precursor 25-hydroxyvitamin D (25OHD) is the generally accepted indicator of vitamin D status. So far, circulating 1,25(OH)_2_D levels have received relatively little attention, except in chronic kidney disease (CKD) patients. This is, at least in part, due to the fact that the half-life of 1,25(OH)_2_D in the circulation is only a few hours and the fact that circulating 1,25(OH)_2_D levels are considered to be tightly regulated within a narrow range [2, 5]. However, besides poor kidney function, diabetes mellitus, high levels of the inflammatory marker C-reactive protein, and high EuroSCORE values (a cardiosurgical risk marker) are also independently associated with low circulating 1,25(OH)_2_D levels [6]. Several studies have demonstrated that circulating 1,25(OH)_2_D is a good predictor of poor outcome in heart failure and sepsis patients [7–10]. Moreover, in diabetic patients, circulating 1,25(OH)_2_D is inversely associated with calcified plaque progression of coronary arteries [11]. Thus, the clinical relevance of circulating 1,25(OH)_2_D is probably underappreciated.

Due to its picomolar concentrations and its lipophilic nature, 1,25(OH)_2_D represents the most difficult challenge of all the steroid hormones to the analytical biochemist with respect to quantification [12]. Until recently, relatively large sample volumes and extensive purification and separation steps were required. Notably, a recent meta-analysis has demonstrated that results of circulating 1,25(OH)_2_D measurement depend on the test procedure used [13]. The differences in 1,25(OH)_2_D measurement limit comparison of results from different laboratories and methods, as well as the development of uniform reference values for circulating 1,25(OH)_2_D levels. Therefore, automatization and standardization are required to improve the reliability of test procedures.

A newly developed automated immunoassay has shown good concordance with measurement by a liquid chromatography tandem mass spectrometry reference method (LC-MS/MS) under standardized conditions [14]. However, recent data by the Vitamin D External Quality Assessment Scheme (DEQAS) have demonstrated that coefficients of variation within specific tests and mean 1,25(OH)_2_D levels between different test procedures can both vary by more than 20% [15].

The present investigation thus aimed to test the comparability of a commercially available LC-MS/MS method with a new and already frequently used automated assay in routine practice settings in the clinical laboratories.

## 2. Materials and Methods

### 2.1. Study Population

Comparison of the two methods was performed with serum samples from patients scheduled for cardiac surgery who agreed to participate in a prospective cohort study. The protocol and characteristics of the study have been published elsewhere [6]. The study was approved by the local ethics committee and was registered at clinicaltrials.gov as NCT02192528. All patients have signed an informed consent.

### 2.2. Laboratory Methods

After drawing of fasting blood samples, specimens were centrifuged and serum aliquots were stored at −80°C until analyzed. In 129 serum samples, we measured circulating 1,25(OH)_2_D by an LC-MS/MS method provided by Immundiagnostik (Bensheim, Germany) and also by an automated immunoassay provided by DiaSorin (Stillwater, MN, USA). The commercially available LC-MS/MS method includes an extraction step with ImmunoTube® columns for purification and separation of 1,25(OH)_2_D from the sample. Detection of 1,25(OH)_2_D was performed with a Waters Quattro Premier XE tandem mass spectrometer according to the provider's instruction sheet. Sample volume was 500 *μ*L. The detection limit of the method is 5.7 pg/mL for 1,25(OH)_2_D_3_ and 12 pg/mL for 1,25(OH)_2_D_2_. Since all 1,25(OH)_2_D_2_ values were below the detection limit (in Germany neither vitamin D_2_ supplements nor vitamin D_2_ medications are used, and foods are only supplemented with vitamin D_3_), only 1,25(OH)_2_D_3_ levels were considered for data analysis (designated 1,25(OH)_2_D). According to the manufacturer, the lower and upper limits of the reference range for adults are 17 and 53 pg/mL. The chemiluminescent immunoassay provided by DiaSorin is a fully automated, modified, 3-step sandwich assay that uses a recombinant fusion protein for capture of the 1,25(OH)_2_D molecule and a murine monoclonal antibody which specifically recognizes the complex formed by the recombinant fusion protein with the 1,25(OH)_2_D molecule. The assay runs on the autoanalyzer LIAISON® XL and does not require an extraction step. Cross-reactivity for 1,25(OH)_2_D_2_ (reference: 1,25(OH)_2_D_3_ = 100%) is 104%. Thus, results are the sum of 1,25(OH)_2_D_2_ and 1,25(OH)_2_D_3_. They were designated 1,25(OH)_2_D. The serum volume required for testing is 75 *μ*L per specimen plus 150 *μ*L dead volume (volume at the bottom of the aliquot tube that cannot be aspirated). Samples may be frozen-thawed four times. The limit of quantitation is 5.0 pg/mL. According to the manufacturer, the lower and upper limits of the expected reference range are 19.9 and 79 pg/mL.

### 2.3. Statistics

The statistical analyses were performed using SPSS version 21.0 (SPSS Inc., Chicago, IL, USA) and MedCalc version 11.6.1.0 (MedCalc Software bvba, Ostend, Belgium). Since data were nonnormally distributed (tested by the Kolmogorov-Smirnov test), results are given as median and interquartile range (IQR) and are graphically presented using box and whisker plots, as well as ogive. The paired-sample Wilcoxon signed-rank test was performed for determination of differences between the analytical tests. The concordance correlation coefficient was applied to evaluate the degree to which pairs of observations fall on the 45° line through the origin [16]. It contains a measurement of precision (Pearson's correlation coefficient *r*) and accuracy [bias correction factor (Cb)] and is calculated as follows: concordance correlation coefficient = *r* × Cb. Pearson's correlation coefficient measures how far each observation deviates from the best-fit line. Cb measures how far the best-fit line deviates from the 45° line through the origin. Moreover, the agreement between methods was assessed by Bland-Altman plot [17] and with Passing-Bablok method [18]. The CUSUM test was used to assess whether residuals were randomly scattered above and below the regression line and did not exhibit any distinct trend. *P* values below 0.05 were considered statistically significant.

## 3. Results

**Figure 1:**
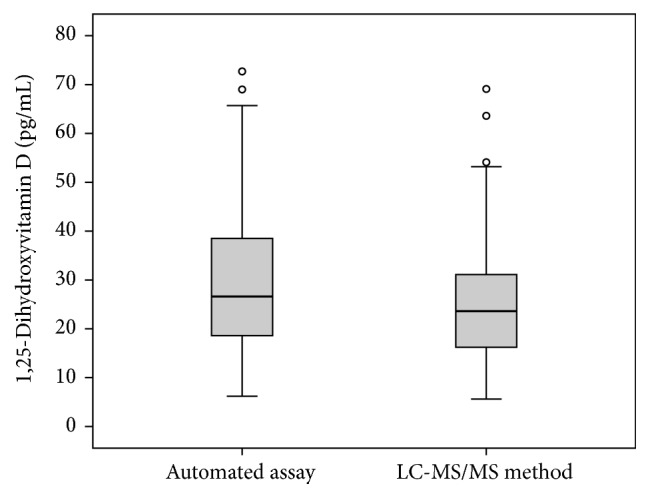
Box and whisker plots showing the distribution of results for the automated assay and the LC-MS/MS method. The central boxes express the upper and lower quartile, and the central lines show the median. The whiskers represent the values below and above the interquartiles, excluding outliers. Outliers (circles) are defined as values that exceed the upper and lower quartile plus or minus 1.5 times the interquartile range.

The 129 patients had a median age of 73 years (IQR: 64–79 years; range: 34 to 89 years), and 32.3% were females. Box and whisker plots showing the distribution of the results of the two methods are given in Figure 1. Median (IQR) 1,25(OH)_2_D concentrations with the automated assay and the LC-MS/MS method were 26.6 pg/mL (18.5–39.0 pg/mL) and 23.6 pg/mL (16.1–31.3 pg/mL), respectively (*P* = 0.001).

**Figure 2:**
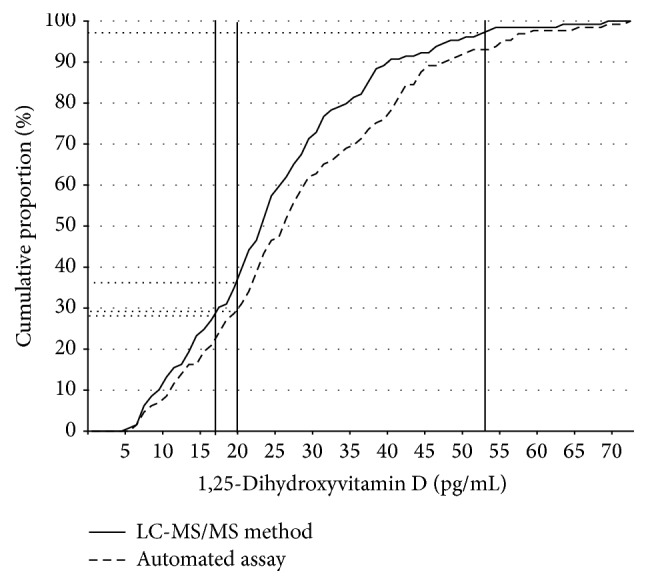
Ogive showing the percentage of specimen with 1,25-dihydroxyvitamin D concentrations below a certain value according to test procedure. The vertical lines mark different cut-off levels for deficient or harmful 1,25-dihydroxyvitamin D concentrations.

Figure 2 shows the percentages of patients below a certain 1,25(OH)_2_D threshold. Using a value of <20 pg/mL as the cut-off for deficient 1,25(OH)_2_D levels, the percentages of patients classified as 1,25(OH)_2_D deficient with the automated assay and the LC-MS/MS method were 28.7% and 36.4%, respectively. However, if the method-specific cut-offs were used (<20 pg/mL for the automated assay and <17 pg/mL for the LC-MS/MS method), the percentages of patients classified as 1,25(OH)_2_D deficient were 28.7% and 27.1% and were thus almost identical for both methods. None of the samples had 1,25(OH)_2_D concentrations above 80 pg/mL. If the method-specific upper limit of the reference range was used, the percentage of patients classified as having high 1,25(OH)_2_D levels was 0% with the automated assay (>79 pg/mL) and 3.1% (>53 pg/mL) with the LC-MS/MS method. However, concordance for the automated method and LC-MS/MS for deficient levels (<20 pg/mL and <17 pg/mL, resp.) was only 62% (38% were deficient according to the automated assay but normal according to the LC-MS/MS method). Concordance for the automated method and LC-MS/MS for adequate levels (20 to 79 pg/mL and 17 to 53 pg/mL, resp.) was 86%.

**Figure 3:**
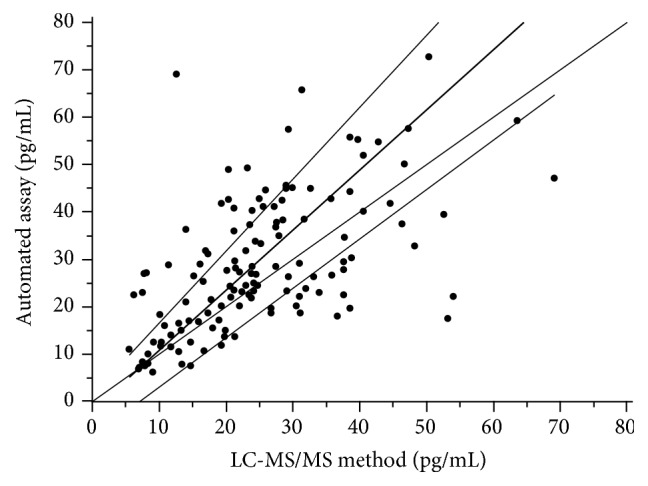
Passing-Bablok regression analysis of the two methods.

Passing-Bablok regression analysis is presented in Figure 3. The concordance correlation coefficient between the two methods was 0.534 (95% CI: 0.406–0.642). The regression equation resulted in an intercept of −1.99 (95% CI: −7.33 to 1.31) and a slope of 1.27 (95% CI: 1.04 to 1.52) for the automated assay. The CUSUM test indicates a significant deviation from linearity (*P* < 0.05).

**Figure 4:**
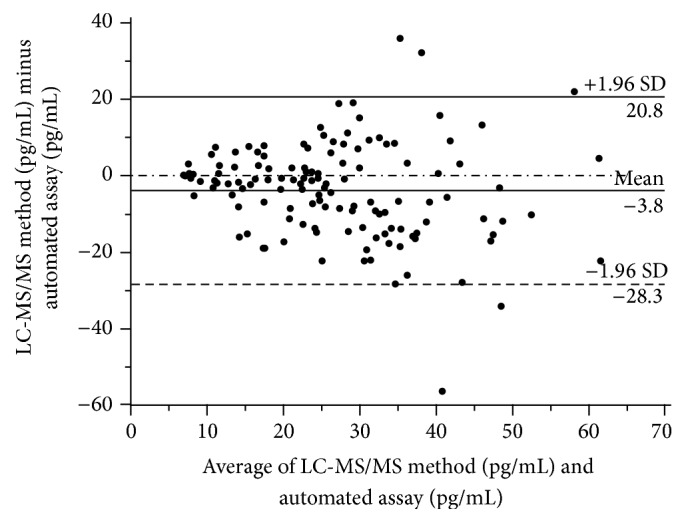
Bland-Altman plot showing the bias between the automated method and the mean of the two methods.

**Figure 5:**
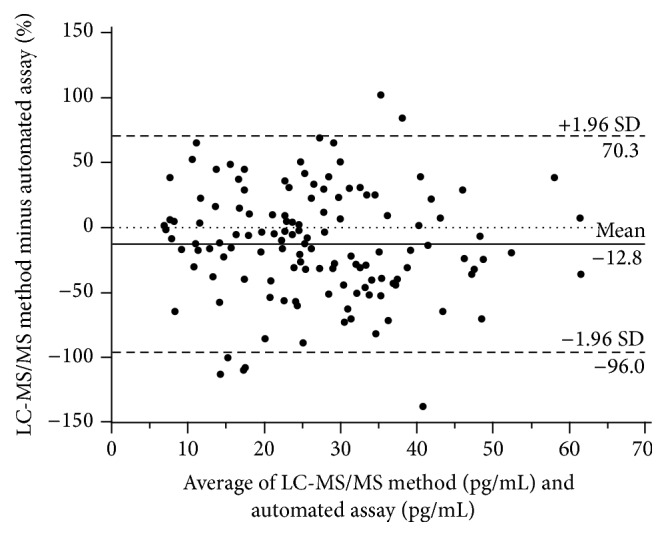
Bland-Altman plot showing the bias between the automated method and the mean of the two methods on a percentage basis.

The mean bias with respect to the mean of the two methods was −3.8 (1.96 SD, −28.3 to 20.8) pg/mL for the LC-MS/MS method minus the automated assay (Figure 4). The limits of agreement, expressed as a percentage of the 1.96 SD of mean between the methods, were very large (Figure 5). Particularly, with the LC-MS/MS method, a large downward deviation from the mean of the two methods was obvious in several samples.

## 4. Discussion

The present study demonstrated significant deviation in 1,25(OH)_2_D concentrations between the two methods, indicating the need for further standardization of 1,25(OH)_2_D measurement. However, results also demonstrate that the percentage of samples classified as “deficient” or “high” in 1,25(OH)_2_D was comparable between the two groups, especially if method-specific cut-offs were used.

1,25(OH)_2_D is a difficult analyte. The commercial availability of an automated immunoassay using low sample volume and not requiring sample extraction as well as direct 1,25(OH)_2_D determination by a commercially available LC-MS/MS test kit are milestones in measuring this analyte. The general reliability of both methods is supported by the fact that the automated assay uses a highly specific recombinant fusion protein for capture of the 1,25(OH)_2_D molecule [14], whereas the LC-MS/MS method uses an extraction step for purification and separation of 1,25(OH)_2_D from the sample, which seems to be crucial for reliable 1,25(OH)_2_D measurement with an LC-MS/MS method [14]. Nevertheless, the two methods show only modest correlation and further standardization is required to improve reliability and comparability of 1,25(OH)_2_D test procedures. Although the percentages of patients with levels below the respective cut-off values were more or less equal, they represent different patients. Therefore, agreement between the two methods may be considered acceptable with respect to 1,25(OH)_2_D quantification and classification when considering results at population level but not from the perspective of an individual patient. This position is supported by the large percentage of deviation of several samples from the mean of the two methods in the present investigation. Other recent comparison studies between the automated DiaSorin method and LC-MS/MS show far better performances [19–21]. Correlation coefficients between the automated method and LC-MS/MS of 0.92 to 0.998 have been reported [19–21]. However, such high correlation coefficients are highly questionable, at least in routine practice settings in clinical laboratories, due to imprecision in sample purification, separation, and measurement. As mentioned before, the DEQAS data have shown substantial differences in the measurement of 1,25(OH)_2_D concentrations among different methods [15]. In detail, the July 2015 evaluation of five different samples revealed in the 63 labs using the automated DiaSorin method mean 1,25(OH)_2_D concentrations of 101.5 pmol/L, 96.1 pmol/L, 91.4 pmol/L, 124.6 pmol/L, and 142.1 pmol/L (to convert into pg/mL, divide by 2.4). The corresponding values for the 13 labs using LC-MS/MS were 100.8 pmol/L, 102.1 pmol/L, 79.1 pmol/L, 112.5 pmol/L, and 130.7 pmol/L, respectively. In addition, considerable variation in 1,25(OH)_2_D concentrations was present among the 13 labs using LC-MS/MS.

Several recent studies on the circulating 1,25(OH)_2_D levels support the assumption that this steroid hormone is an important predictor of certain clinical outcomes [7–11]. Moreover, a recent meta-analysis of randomized controlled trials has identified several gaps in the present knowledge with respect to the circulating 1,25(OH)_2_D levels [13]. These gaps include (i) incomplete data about the effect of activated vitamin D administration on the circulating 1,25(OH)_2_D levels, (ii) inadequate data about the effect of native vitamin D on the circulating 1,25(OH)_2_D levels (including the influence of vitamin D dosage and initial 25OHD levels on the circulating 1,25(OH)_2_D), (iii) the question of whether or not in CKD patients high dose oral vitamin D supplementation can be a substitute for activated vitamin D with respect to the effect on the circulating 1,25(OH)_2_D levels, and (iv) the influence of diseases and physical activity on the circulating 1,25(OH)_2_D levels. Thus, efforts towards standardization should be enforced in order to achieve comparability between studies.

Theoretically, there may be several causes for systematic differences and random errors between the automated assay and the LC-MS/MS method. First, the LC-MS/MS method may underestimate the true 1,25(OH)_2_D concentration. Note that in all samples 1,25(OH)_2_D_2_ levels were below the detection limit of the LC-MS/MS method, which was 12 pg/mL. However, even in individuals not taking supplements containing vitamin D, food-based 25OHD_2_ levels are on average 1.4 ng/mL (10th–90th percentile: 0.68 to 2.48 ng/mL) [22]. Thus, in most individuals small amounts of 1,25(OH)_2_D_2_ should also be present in the circulation, but these levels may be below the detection limit of the LC-MS/MS method. Since the automated method measures both 1,25(OH)_2_D_2_ and 1,25(OH)_2_D_3_, this may, at least in part, explain why total 1,25(OH)_2_D levels were, on average, 3 pg/mL higher than those measured with the LC-MS/MS method. Second, although sample extraction for the LC-MS/MS method is capable of eliminating interfering matrix components [23], residual effects leading to ion suppression cannot be definitively excluded. This may explain why on a percentage basis the LC-MS/MS method showed a large downward deviation in several samples of different concentrations. Third, unknown cross-reacting substances may lead to increased 1,25(OH)_2_D with the automated assay.

With respect to 25OHD measurement, it has already been demonstrated that standardization is absolutely necessary to obtain reliable and comparable results [24, 25]. In the European Commission-funded ODIN project (food-based solutions for optimal vitamin D nutrition and health through the life cycle) [26], measures will be taken to compare and standardize results of already existing data on circulating 25OHD levels in Europe, obtained with different methods. Similar to 25OHD measurement, an international program is necessary to standardize 1,25(OH)_2_D measurement across methods and manufacturers.

Our study has some limitations. First, the study population was limited to cardiac surgical patients and did not include serum from patients with hypercalcemia due to vitamin D intoxication or other causes, which is an important reason to measure 1,25(OH)_2_D. Second, we did not compare assays for resistance to interference for substances such as paricalcitol [27] or very high levels of 25OHD_2_ and 25OHD_3_. Third, we did not use a reference measurement procedure and reference materials, which complicates the interpretation of method accuracy. However, it is noteworthy that the present investigation was not performed to assess assay accuracy but to compare two commercially available methods in routine practice settings in the clinical laboratory.

## 5. Conclusions

Both commercial methods are important innovations for the measurement of this difficult analyte. Nevertheless, the correlation between the two methods is only modest, at least in the routine practice settings in clinical laboratories, and further standardization is required to improve reliability and comparability of 1,25(OH)_2_D test procedures.
